# Supplementary material for: Development and External Validation of Partial Proportional Odds Risk Prediction Models for Cancer Stage at Diagnosis among Males and Females in Canada
Source: Cancers (Basel). 2023 Jul 8;15(14):3545. doi: 10.3390/cancers15143545 (PMC10377619; doi:10.3390/cancers15143545)
Supplement: Supplementary file 1 [file cancers-15-03545-s001.zip › cancers-2414503-supplementary.pdf]

Supplemental materials: Biziaev et al. Development and external validation of partial proportional odds risk prediction models for cancer stage at diagnosis among males and females in Canada

Supplemental Table S1. Variable definitions and revised response coding used from Alberta's Tomorrow Project and British Columbia Generations Project cohorts

	**ATP (n= 2112)**		**BCGP (n = 855)**		
**Variable**	**Data Source**	**Response Options**	**Data Source**	**Response Options**	**Coding**
Cancer stage	ACR administrative data linkage		BCCR administrative data linkage		I/II/III/IV
Age at cancer diagnosis (years and months)	ACR administrative data linkage		BCCR administrative data linkage		Real number value in the analysis
Employment	HLQ	7 options	HLCQ	8 options	Collapsed to full or part-time/other
Family history of heart attack	HLQ	Respondent chooses from a list of diagnoses per immediate family member	HLCQ	Respondent chooses from a list of diagnoses per immediate family member	Yes/no
Have ever had mammogram test	HLQ	Q: Have you ever had a mammogram test (a breast x-ray)?	HLCQ	Q: When was the last time you had a mammogram?.<br><br>7 options, including never	Yes/no
Have ever had PSA test	HLQ	Q: Have you ever had a PSA blood test? (This is a specific test ordered by a doctor to test men for prostate cancer.)	HLCQ	Q: When was the last time you had a PSA blood test? A PSA test is a specific blood test ordered by a doctor to test men for prostate cancer. 7 options including never	Yes/no
Have ever had sigmoidoscopy/ colonoscopy	HLQ		HLCQ	Q: When was the last time you had a colonoscopy?<br><br>Q: When was the last time you had a sigmoidoscopy?	Yes/no
Number of pregnancies	HLQ	Q: How many times have you been pregnant?	HLCQ	Q: How many times have you been pregnant, including live births, stillbirths,	Integer

				spontaneous miscarriages, or therapeutic abortions?	
Have ever had hysterectomy	HLQ	Q: Did you ever have a hysterectomy? (An operation to have your uterus or womb removed)	HLCQ	Q: Have you ever had a hysterectomy (an operation to have your uterus or womb removed)?	Yes/no
Type of smoker	HLQ, Derived by ATP	Multiple (>20) questions	HLCQ	Multiple (18) questions	Collapsed to current smoker/past smoker/never smoker

ACR, Alberta Cancer Registry; ATP, Alberta's Tomorrow Project; BCCR, British Columbia Cancer Registry; BCGP, British Columbia Generations Project; HLQ, Health and Lifestyle Questionnaire; HLCQ, Health and Lifestyle Core Questionnaire

Table S2. Aggregated cancer sites for category "other cancers" by cohort

**Cancer sites other than breast, lung, prostate, and colorectal cancer over 5%**				
	**ATP**		**BCGP**	
	**Cancer Cases from the Alberta Cancer Registry (n)**	**Percentage (%)**	**Cancer Cases from the BC Cancer Registry (n)**	**Percentage (%)**
Female Genital Organs	168	24%	63	25%
Digestive Organs	153	22%	72	29%
Hematopoietic & Reticuloendothelial Systems	107	16%	62	25%
Urinary Tract	89	13%	–	–
Melanoma	65	9%	–	–
Thyroid & Other Endocrine Glands	42	6%	–	–
Head & Neck	39	6%	18	7%
Total	688	100%	252	100%

The percentages do not sum to 100%, due to that fact that only cancer sites other than breast, lung, prostate, and colorectal cancer having more than 5% among total cases of all the other cancer sites were included.

Supplemental Table S3. Participant characteristics of Alberta's Tomorrow Project and British Columbia Generations Project cohorts

	ATP Development		BCGP Validation	
**Characteristic**	**Males (n = 903) n (%)**	**Females (n = 1209) n (%)**	**Males (n = 298) n (%)**	**Females (n = 557) n (%)**
Cancer stage at diagnosis				
I	196 (21.7)	521 (43.1)	70 (23.5)	266 (47.8)
II	369 (40.9)	311 (25.7)	115 (38.6)	129 (23.2)
III	150 (16.6)	207 (17.1)	59 (19.8)	96 (17.2)
IV	188 (20.8)	170 (14.1)	54 (18.1)	66 (11.8)
Ethnicity‡				
Caucasian	875 (96.9)	1176 (97.3)	281 (94.3)	490 (88.0)
Non-Caucasian	28 (3.1)	33 (2.7)	17 (5.7)	67 (12.0)
Education†‡				
High school diploma	296 (32.8)	420 (34.7)	63 (21.1)	144 (25.9)
Some university	399 (44.2)	542 (44.8)	96 (32.2)	225 (40.4)
University degree	208 (23.0)	247 (20.4)	139 (46.6)	188 (33.8)
Married or common in-law				
Yes	755 (83.6)	879 (72.7)	246 (82.6)	389 (69.8)
No	148 (16.4)	330 (27.3)	52 (17.4)	168 (30.2)
Employment†‡				
Full or part-time	603 (66.8)	722 (59.7)	125 (41.9)	290 (52.1)
Other	300 (33.2)	487 (40.3)	173 (58.1)	267 (47.9)
Annual household Income ($)†‡				
< 50K	289 (32.0)	534 (44.2)	86 (28.9)	196 (35.2)
50 - 100K	404 (44.7)	488 (40.4)	120 (40.3)	216 (38.8)
> 100K	210 (23.3)	187 (15.5)	92 (30.9)	145 (26.0)
Family history of diabetes‡				
Yes	241 (26.7)	349 (28.9)	73 (24.5)	194 (34.8)
No	662 (73.3)	860 (71.1)	225 (75.5)	363 (65.2)
Family history of heart attack				
Yes	310 (34.3)	477 (39.5)	121 (40.6)	218 (39.1)
No	593 (65.7)	732 (60.5)	177 (59.4)	339 (60.9)
Family history of stroke†‡				
Yes	189 (20.9)	306 (25.3)	85 (28.5)	175 (31.4)
No	714 (79.1)	903 (74.7)	213 (71.5)	382 (68.6)
Family history of breast cancer‡				
Yes	119 (13.2)	179 (14.8)	37 (12.4)	107 (19.2)
No	784 (86.8)	1030 (85.2)	261 (87.6)	450 (80.8)
Family history of colorectal cancer				
Yes	94 (10.4)	120 (9.9)	41 (13.8)	63 (11.3)

No	809 (89.6)	1089 (90.1)	257 (86.2)	494 (88.7)
Family history of prostate cancer				
Yes	101 (11.2)	95 (7.9)	50 (16.8)	56 (10.1)
No	702 (77.7)	1114 (92.1)	248 (83.2)	501 (89.9)
Family history of lung cancer†‡				
Yes	82 (9.1)	120 (9.9)	54 (18.1)	82 (14.7)
No	821 (90.9)	1089 (90.1)	244 (81.9)	475 (85.3)
Family history of all cancer†‡				
Yes	515 (57)	703 (58.1)	208 (69.8)	390 (70.0)
No	388 (43)	506 (41.9)	90 (30.2)	167 (30.0)
Family history of other†‡				
Yes	311 (34.4)	460 (38.0)	59 (19.8)	131 (23.5)
No	592 (65.6)	749 (62.0)	239 (80.2)	426 (76.5)
Have ever had blood stool test†‡				
Yes	337 (37.3)	456 (37.7)	207 (69.5)	330 (59.2)
No	566 (62.7)	753 (62.3)	91 (30.5)	227 (40.8)
Have ever had sigmoidoscopy or colonoscopy†‡				
Yes	212 (23.4)	322 (26.6)	149 (50.0)	252 (45.2)
No	691 (76.5)	887 (73.4)	149 (50.0)	305 (54.8)
Have ever had PSA blood test†				
Yes	389 (43.1)	-	254 (85.2)	-
No	514 (56.9)	-	44 (14.8)	-
Have ever had mammogram‡				
Yes	-	1033 (85.4)	-	521 (93.5)
No	-	176 (14.6)	-	36 (6.5)
Have ever had pap smear test‡				
Yes	-	1141 (94.4)	-	540 (96.9)
No	-	68 (5.6)	-	17 (3.1)
Have ever had oophorectomy‡				
Yes	-	102 (8.4)	-	69 (12.4)
No	-	1107 (91.6)	-	476 (85.5)
Missing	-		-	12 (0.2)
Have ever had hysterectomy				
Yes	-	298 (24.6)	-	126 (22.6)
No	-	911 (75.4)	-	431 (77.4)
Have ever been pregnant‡				
Yes	-	1091 (90.2)	-	451 (81.0)

No	-	118 (9.8)	-	106 (19.0)
Have ever had hormone replacement therapy for menopause				
Yes	-	490 (40.5)	-	239 (42.9)
No	-	719 (59.5)	-	318 (57.1)
Smoking status†‡				
Non-smoker	291 (32.2)	474 (39.2)	139 (46.6)	280 (50.3)
Past smoker	417 (46.2)	444 (36.7)	142 (47.7)	244 (43.8)
Current smoker	195 (21.6)	290 (24.0)	17 (5.7)	33 (5.9)
Self-perceived health†‡				
Excellent/very good	367 (40.6)	555 (45.9)	182 (61.1)	373 (67.0)
Good/fair	425 (47.1)	505 (41.8)	110 (36.9)	177 (31.8)
Poor	111 (12.2)	149 (12.3)	2 (0.7)	4 (0.7)
	Median (Q1-Q3)	Median (Q1-Q3)	Median (Q1-Q3)	Median (Q1-Q3)
Age at enrollment (years) †‡	57 (50-63)	55 (47-62)	64 (60-67)	60 (53-65)
Age at diagnosis (years) †‡	65 (58-70)	63 (55-69)	67 (63-70)	64 (56-68)
Age at first menstruation (years)		13 (12-13)		13 (12-14)
Missing				21
Alcohol consumption (servings/week) †‡	5.0 (1.1-9.3)	1.0 (0.2-3.4)	0.0 (0.0-6.0)	0.0 (0.0-4.0)
Fruit and vegetable intake (servings/week) †‡	4.1 (2.6-5.5)	4.4 (2.9-6.0)	5.0 (3.0-7.0)	5.0 (4.0-7.0)
Comorbidity (0-7)†	0 (0-1)	1 (0-1)	1 (0-1)	1 (0-2)
Number of pregnancies‡	-	3 (2-4)	-	2 (1-3)

†Statistically significant difference for males between datasets $p < 0.05$; ‡ Statistically significant difference for females between datasets $p < 0.05$; Comorbidity index: range (0-7), Q1 = first quartile, Q3 = third quartile
